# Management of adverse renal events related to alemtuzumab treatment in multiple sclerosis: a Belgian consensus

**DOI:** 10.1007/s13760-017-0864-x

**Published:** 2017-11-30

**Authors:** Ben Sprangers, D. Decoo, D. Dive, A. Lysandropoulos, L. Vanopdenbosch, C. Bovy

**Affiliations:** 1 Department of Nephrology, University Hospitals Leuven, Herestraat 49, 3000 Leuven, Belgium; 2 Laboratory for Experimental Transplantation, Department of Microbiology and Immunology, University of Leuven, Leuven, Belgium; 3 Neurology Department, AZ Alma, Gentse Steenweg 132, 8340 Sijsele, Belgium; 4 Service de Neurologie, CHU Liège, Esneux, Belgium; 5 Neuroimmunology, MS Unit, Neurology Department, CUB, Hôpital Erasme, Route de Lennik 808, 1070 Brussels, Belgium; 6 Department of Neurology, AZ Sint Jan Brugge Oostende, Ruddershove 10, 8000 Brugge, Belgium; 7 Service de Néphrologie, CHU Sart-Tilman, B35, 4000 Liège, Belgium

**Keywords:** Multiple sclerosis, Alemtuzumab, Renal adverse event, Autoimmunity, Consensus guideline

## Abstract

Alemtuzumab is a humanized monoclonal antibody indicated for the treatment of adult patients with relapsing–remitting multiple sclerosis with active disease. Multiple sclerosis (MS) patients treated with alemtuzumab are at increased risk for autoimmune adverse events (thyroid disorders, immune thrombocytopenia, and renal disease). The use of alemtuzumab has been associated with the development of renal immune-mediated adverse events in 0.3% of patients in clinical trials in MS, which generally occurred within 39 months of the last administration. Both anti-GBM disease and membranous nephropathy have been associated with the use of alemtuzumab. Early detection is necessary to allow for early diagnosis and prevent adverse renal and patient outcomes. Through the implementation of the risk minimization measures, patients can be diagnosed, and treated if needed, early allowing for generally favorable outcomes. This important goal can be reached through health care professional and patient education, careful analysis of the monthly lab tests, and close collaboration between the patient, neurologist, and the nephrologist. This article presents the consensus of Belgian MS specialists and nephrologists on the practicalities of diagnosis, management, and treatment of alemtuzumab-associated renal adverse events based on good clinical practice.

## Introduction

Alemtuzumab is a humanized monoclonal antibody approved in more than 60 countries for the treatment of multiple sclerosis (MS), and is marketed under the name Lemtrada^®^. Within the European Union, alemtuzumab is indicated for the treatment of adult patients with relapsing–remitting multiple sclerosis (RRMS) with active disease defined by clinical or imaging features. In clinical trials, alemtuzumab demonstrated superior efficacy compared to high-dose subcutaneous (SC) interferon beta-1a (IFNb-1a) in both treatment-naïve patients and in those with inadequate response to prior therapy, with a consistent and manageable safety and tolerability profile [1]. The most recent efficacy data over 6 years on clinical and MRI lesion activity as well as on brain volume loss suggest that alemtuzumab may provide a unique treatment approach for RRMS patients, offering durable efficacy in the absence of continuous treatment [2].

MS patients treated with alemtuzumab are at increased risk for autoimmune adverse events (AEs) (thyroid disorders, ITP, and renal disease). Two major types of autoimmune renal diseases have been associated with the use of alemtuzumab: membranous nephropathy and anti-glomerular basement membrane disease (anti-GBM disease) [3, 4]. Especially, in anti-GBM disease, early diagnosis is necessary to prevent adverse renal and patient outcomes. The Lemtrada^®^ Risk Management Plan was put in place to ensure early detection of symptoms or signs of autoimmune disease, with the aim of minimizing the impacts of alemtuzumab-associated renal effects while maximizing the clinical benefits of the drug with respect to the treatment of RRMS. Renal surveillance includes monthly measurement of serum creatinine and urine analysis with microscopy (detection of red blood cells and proteinuria). In addition, the patient and treating physician are educated to recognize symptoms potentially related to renal disease, such as edema, discolored urine, and hemoptysis [5, 6]. This monitoring has to be performed for 48 months after the last alemtuzumab administration Nephrology consultation is recommended in the management of nephropathies. This article presents the consensus of Belgian MS specialists and nephrologists on the practicalities of diagnosis, management, and treatment of Lemtrada-associated renal AEs based on good clinical practice.

## Alemtuzumab and autoimmune renal disease

Autoimmune AEs were detected in MS patients treated with alemtuzumab in clinical trials [7]. The 6-year follow-up data of the CARE-MS studies were presented at ECTRIMS 2016 and showed the following frequencies: 39% of alemtuzumab-treated patients experienced an autoimmune thyroid disorder, 2.6% an immune thrombocytopenic purpura, and 0.2% (two cases) an autoimmune renal disease [2]. In post-marketing use through February 2017, 13,000 patients have been treated worldwide with alemtuzumab for MS and the frequency for anti-GBM disease and membranous nephropathy was estimated at 0.13% [8]. Post-marketing frequencies are not directly comparable to clinical trial incidences because of differences in ascertainment methodology and follow-up duration, and limitations of post-marketing reporting.

### Anti-glomerular basement membrane (anti-GBM) disease

Anti-GBM disease is a rare renal disease caused by the presence of antibodies directed against an antigen located in the glomerular basement membrane (noncollagenous domain 1 of the α3 chain of type IV collagen (α3[IV]NC1), resulting in rapidly progressive glomerulonephritis with crescent formation with or without concomitant pulmonary symptoms (hemoptysis, shortness of breath, and cough) [9, 10]. Crescents are defined as two or more layers of proliferating cells in Bowman’s space and are a hallmark of inflammatory glomerulonephritis and a histologic marker of severe glomerular injury. Goodpasture’s disease is defined as the presence of glomerulonephritis and pulmonary hemorrhage in the presence of anti-GBM antibodies.

Anti-GBM disease affects approximately 0.5–1.0 patients per million in the general population. This disease represents around 20% of cases of rapidly progressive glomerulonephritis and is found in less than 3% of all kidney biopsies for any reason [11, 12]. Anti-GBM disease occurs in older children and adults without age preference. Younger patients more often have concurrent pulmonary involvement, than older patients [13, 14]. Although anti-GBM disease is most often idiopathic, several triggering factors have been proposed, e.g., infection, smoking, hydrocarbons, smoked cocaine, urinary tract obstruction, and lithotripsy [9, 15–18]. A genetic predisposition to anti-GBM disease has been reported: increased risk associated with HLA-DR15 and DR4 and decreased risk associated with HLA-DR1 and DR7 [19–22]. HLA-DR15 is also associated with an increased risk of MS.

Clinically, patients affected by anti-GBM disease can present with diverse symptoms [general symptoms, edema, hypertension, pulmonary symptoms (dyspnea, hemoptysis)]. General symptoms such as fever, malaise, anorexia, weight loss, and arthralgia may occur but are generally only mild. The outcome of anti-GBM disease is determined by the renal function, the need for dialysis at presentation, and the number of glomeruli affected by crescent formation determined post biopsy. Without immunosuppressive treatment, death or dialysis ensues in more than 90% of patients with anti-GBM disease [11]. A recent report confirmed oligoanuria as the strongest negative predictor of renal and patient survival. Furthermore, the percentage of glomerular crescents was the only pathologic parameter associated with poor renal outcome in anti-GBM disease [23].

The treatment of anti-GBM disease consists of a combination of plasmapheresis, corticosteroids, and cyclophosphamide. Plasmapheresis will result in a rapid decline of anti-GBM antibodies in the circulation, while prednisone and cyclophosphamide are administered to halt antibody production. Almost half of anti-GBM patients will benefit from this treatment by not progressing to dialysis-dependency and survive [24, 25]. Renal recovery is most likely in patients without oligoanuria at the time of diagnosis. Renal recovery is unlikely in patients with 100% of glomeruli affected on biopsy and with early dialysis-dependency. Early initiation of therapy is most important to optimize renal outcome and long-term prognosis [13].

### Membranous nephropathy (MN)

Membranous nephropathy (MN) is the most common cause of nephrotic syndrome in adults. In this disease, immunoglobulins (IgG) and complement factors deposit in the subepithelial area of the glomerular basement membrane (GBM); between the GBM and the visceral epithelial cells (podocytes). Podocytes are highly specialized cells responsible for the protein sieving of the glomerular barrier. Podocytes form interdigitating foot processes covering the GBM. Foot processes are connected through a scale-like protein complex called the “slit-diaphragm”. The presence of antibodies and complement fractions in contact with the podocytes leads to their dedifferentiation with foot process effacement and fusion. Consequently, abnormal amounts of protein pass through the glomerular filter from the vascular compartment into the urine. MN symptoms include edema, hypertension, and lab abnormalities: elevated urinary protein, low serum albumin and protein, and dyslipidemia with or without abnormal glomerular filtration rate.

MN may be primary or secondary in nature. Secondary membranous nephropathy can be seen in the context of autoimmune diseases such as systemic lupus erythematosus (class V according to the ISN/RPS classification) or, more rarely, be associated with infectious diseases (hepatitis B, C, parasites, etc.), drugs and toxins (gold, penicillamine, NSAID), or with malignancies (mainly solid tumors, and more common in patients older than 65 years). In 70% of the cases, MN is primary or idiopathic (pMN). In 2009, the group of David Salant identified the most important antigen involved in the pathogenesis of pMN, the M-type phospholipase A2 receptor (PLA2-R) [26]. This receptor is overexpressed on the membrane of podocytes and may elicit an auto-immunization resulting in the formation of mainly IgG4 anti-PLA_2_R antibodies and the subsequent development of membranous nephropathy. Anti-PLA_2_R antibodies can be detected in 70–80% of patients with pMN. In 2014, another podocyte antigen was reported to be involved in the pathogenesis of pMN: Thrombospondin type-1 domain containing 7A (THSD7A). This antibody is found in approximately 10% of pMN [27]. For the remaining 10–20% of patients with pMN, the responsible antigen has not yet been identified.

The outcome for patients with MN is difficult to predict. Three patterns of clinical evolution have been identified in patients with MN. About one-third will experience spontaneous remission, one-third will remain stable with persisting nephrotic syndrome with no progression to renal failure, and one-third will experience progressive kidney function deterioration necessitating renal replacement therapy (dialysis, renal transplant). Factors that predict poor prognosis (high likelihood of progression to end-stage renal disease) are decreased renal function at diagnosis and heavy proteinuria (> 8 g/day) [28–34]. In addition, the titer of anti-PLA_2_R antibodies has been reported to be a predictor of outcome [35–37].

The diagnosis and treatment of MN are not a nephrological emergency. If MN is considered secondary, the underlying cause should be treated/removed. First line treatment in all MN patients is symptomatic, anti-proteinuric therapy consisting of ACE inhibitors or antagonists of type II angiotensin receptors in combination with salt restriction and, if necessary, diuretics. Immunosuppression is to be considered for patients with persistent nephrotic syndrome and proteinuria above 4 g/day after 6 months of anti-proteinuric therapy and/or disabling or life-threatening symptoms related to nephrotic syndrome (thromboembolic events), and/or patients in whom serum creatinine has risen by 30% or more within 6 months [38]. The recommended immunosuppressive treatment consists of the modified Ponticelli scheme consisting of three 2-monthly cycles of 1 month IV and then oral steroids followed by 1 month oral alkylating agents (cyclophosphamide rather than chlorambucil). Continuous use of oral cyclophosphamide may also be an option. As an alternative, calcineurin inhibitors, cyclosporine + corticosteroid, or tacrolimus monotherapy [13] can be used. In recent years, several groups have reported promising results using rituximab in the treatment of MN [39].

## Nephropathies in the context of alemtuzumab treatment

Anti-GBM disease following alemtuzumab therapy in multiple sclerosis (MS) patients was first reported in 2006 [40]. The first two patients diagnosed with anti-GBM disease related to alemtuzumab treatment both developed to end-stage renal disease, highlighting the necessity to actively monitor for signs of renal disease and allow for early diagnosis and treatment. Subsequently, there has been intense renal surveillance during almost all clinical trial and non-trial treatments resulting in substantially complete documentation of, to date, seven cases of nephropathy in total [41–46]. While drug-related nephropathies are well described, and several mechanisms are recognized [46], alemtuzumab-related nephropathies appear to be a renal manifestation of a general increased tendency of autoimmunity following alemtuzumab treatment. De novo autoimmunity may relate to the proposed mechanism of action of alemtuzumab, i.e., lymphocyte depletion and repopulation. The antibody testing to allow distinction between primary and secondary membranous nephropathy (PLA2-R and THSD7A antibody detection) has not been performed in the patients treated with alemtuzumab. At this moment, we cannot definitely classify these cases of membranous nephropathy as either primary or secondary.

## Management of autoimmune renal AEs in the context of alemtuzumab treatment

Early detection and treatment of nephropathy may decrease the risk of poor outcomes. To minimize the risk of severe disease, regulatory authorities in many countries have approved the marketing of Lemtrada^®^ with the implementation of a comprehensive risk minimization plan that includes health care professional (HCP) and patient education and monthly blood and urine analysis monitoring for 48 months after the last alemtuzumab infusion. In addition, during and after this period of time, testing should be performed based on clinical findings suggestive of nephropathies [6].

The practical recommendations in this paper propose a sound approach in case a renal AE is suspected in an alemtuzumab-treated RRMS patient.

Before starting treatment with alemtuzumab: baseline evaluation of renal status and risk of AE.

As stated in the summary of product characteristics (SmPC), serum creatinine levels and urinalysis with microscopy should be obtained prior to initiation of treatment [6]. Furthermore, our consensus recommendation is to perform a determination of urinary protein content before the initiation of alemtuzumab. Abnormalities identified at this time should be discussed with a nephrologist to see whether additional diagnostic evaluation is required. Whether alemtuzumab can be administered in patients with active glomerular disease has not been investigated and should be discussed on an individual basis.

Once alemtuzumab has been administered: monitoring of renal status. The SmPC clearly defines which tests ought to be done and at what frequency: serum creatinine levels and urinalysis with microscopy should be obtained at monthly intervals thereafter for 48 months after the last infusion. The observation of clinically significant changes (i.e., an increase of 30% or more) from baseline in serum creatinine, unexplained hematuria, and/or proteinuria and symptoms such as edema, should prompt further evaluation for nephropathy including immediate referral to a specialist [6]. It is important to note that small increases in serum creatinine (possibly within the normal range) can correspond to important changes in renal function. Therefore, we consider a relative change of 30% in serum creatinine compared to baseline creatinine being significant.

In the next six sections, different scenarios based on abnormal lab tests and/or symptoms are presented together with our consensus recommendations for the appropriate actions to be taken. Before discussing the different scenarios, we want to stress that any sign of pulmonary involvement of anti-GBM disease (haemoptysis, exertional dyspnoea) warrants immediate contact with a physician and referral to the emergency room and/or evaluation by a nephrologist the same day. Pulmonary involvement has not been seen with alemtuzumab-related anti-GBM disease in clinical trials with alemtuzumab.

### Scenario 1: Isolated microscopic hematuria

**Fig. 1:**
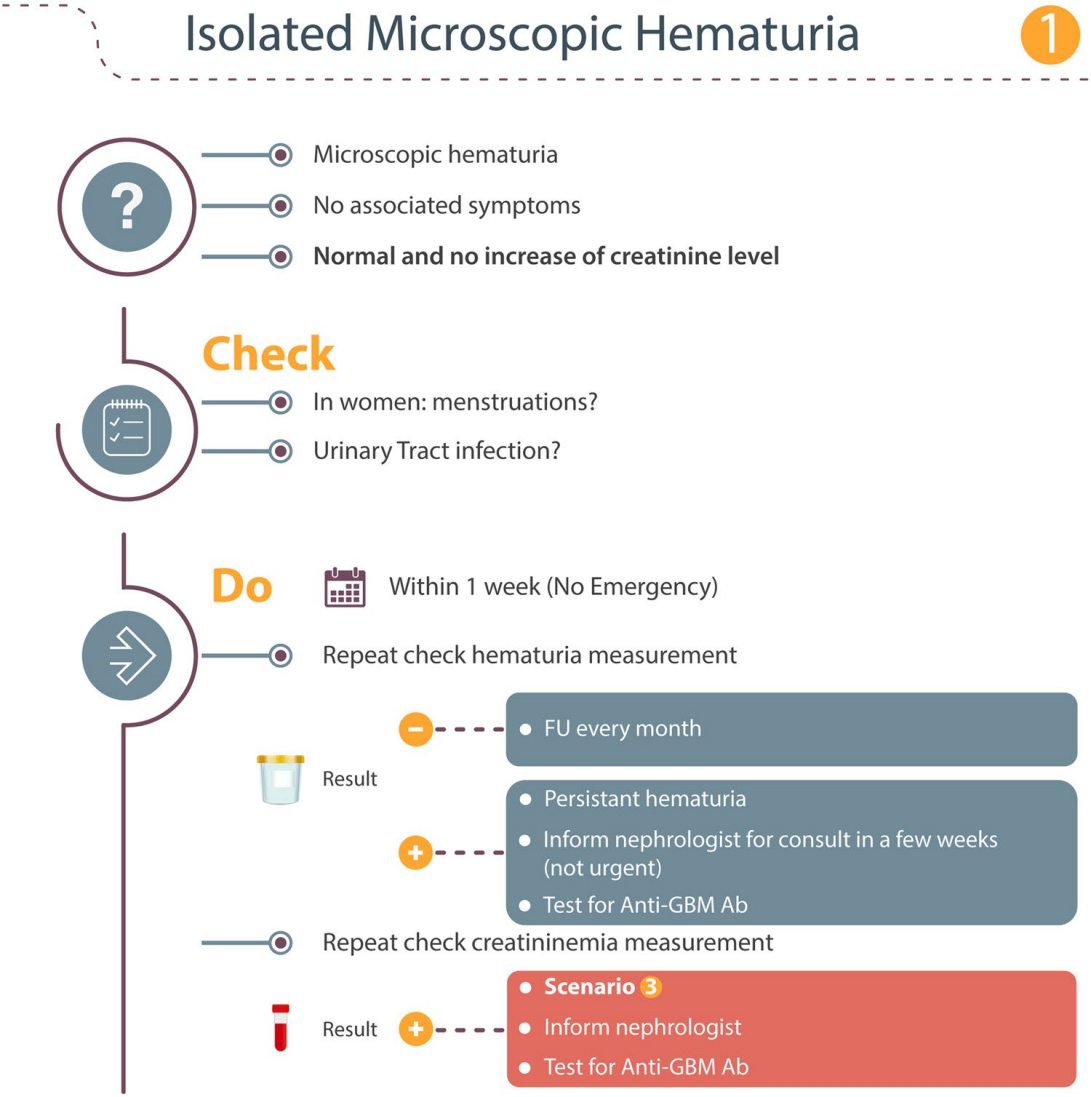
Isolated microscopic hematuria

This will probably be the most frequent scenario (Fig. 1). It is important to note that anti-GBM disease can have a subacute course, so isolated microscopic hematuria can be the initial sign of alemtuzumab-related anti-GBM disease. The first action with this finding is to check for the presence of menstruation in female subjects or a urinary tract infection (UTI). When hematuria is microscopic, urine analysis should be repeated weekly to determine whether hematuria persists. When hematuria has disappeared and serum creatinine remains normal at that time, monthly follow-up should be resumed and no further action is required. In case hematuria persists even in association with a normal serum creatinine, the patient should be referred to a routine nephrology consultancy as soon as possible and a test for anti-GBM antibodies should be carried out. When hematuria is associated with an increased serum creatinine, a nephrologist should be contacted immediately and an anti-GBM antibody test should be carried out. This situation is described in more detail in scenario 3.

### Scenario 2: Isolated gross hematuria

**Fig. 2:**
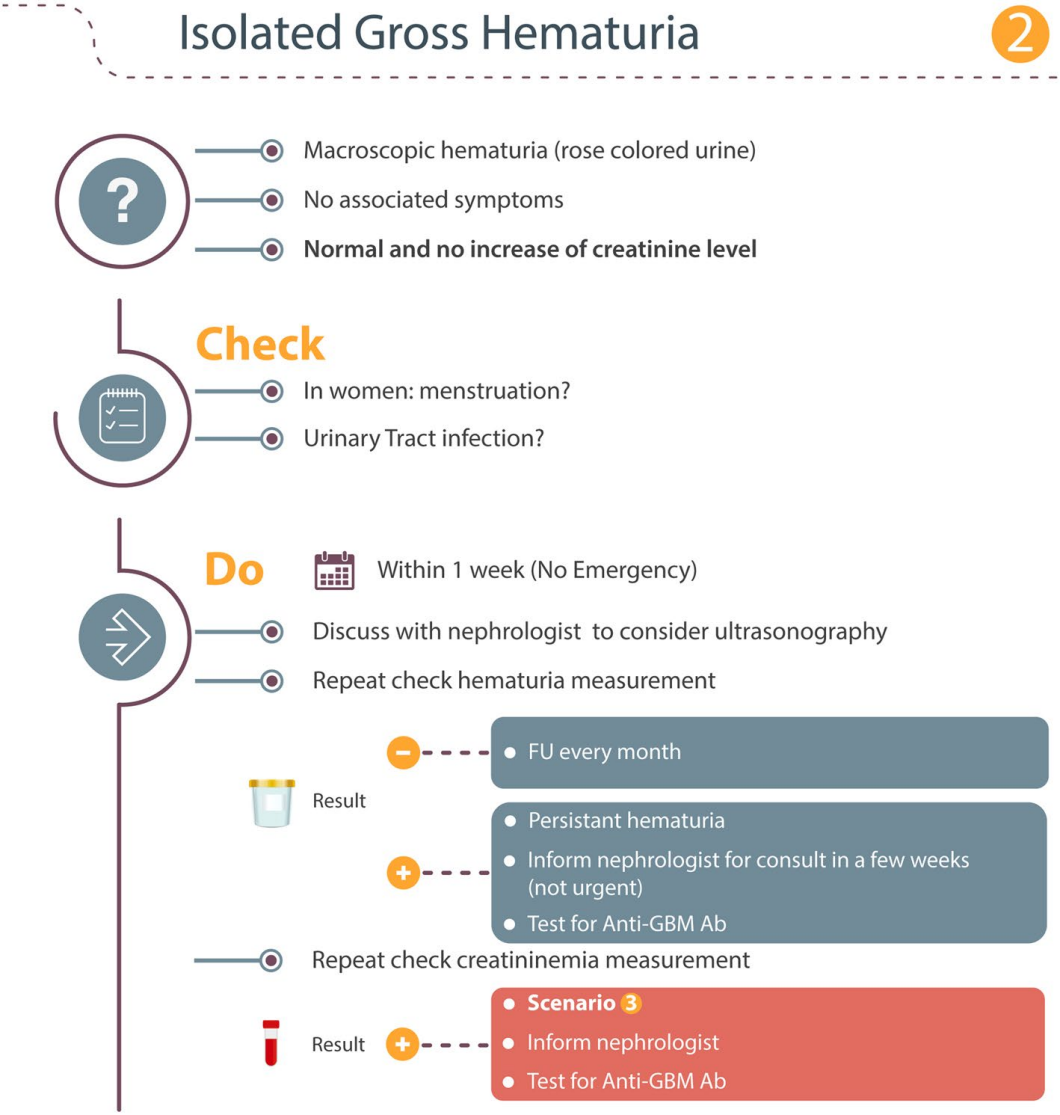
Isolated gross hematuria

If the patient notices a change in their urine color, they must immediately inform their neurologist/GP who should carry out a non-scheduled blood and urine analysis (Fig. 2). Other causes of red discoloration of the urine should be excluded. Gross hematuria is associated with a risk for bladder obstruction because of blood clots and the occurrence of gross hematuria should, therefore, be discussed with a nephrologist to decide whether an urgent ultrasound examination of the urinary tract should be carried out. Otherwise, the same procedure as described in scenario 1 should be followed.

### Scenario 3: Hematuria and elevated serum creatinine! → emergency

**Fig. 3:**
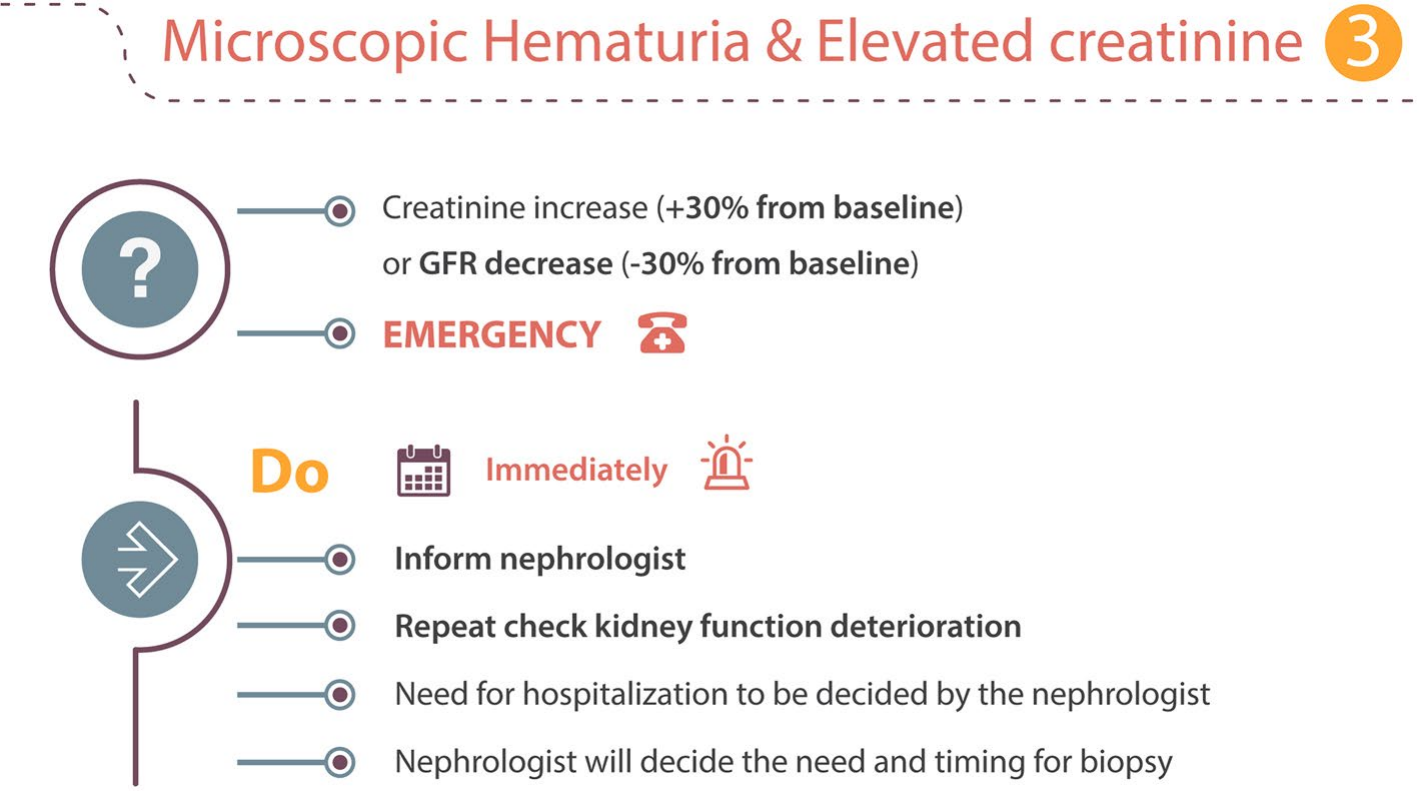
Microscropic hematuria and elevated serum creatinine

A significant increase of serum creatinine (+ 30% from baseline) or a decrease in the glomerular filtration rate (GFR) should prompt immediate action (Fig. 3). Immediate discussion with a nephrologist and biochemical retesting is required. The nephrologist will decide the need for immediate consultation and hospitalization, and whether a kidney biopsy is indicated.

### Scenario 4: Isolated proteinuria

**Fig. 4:**
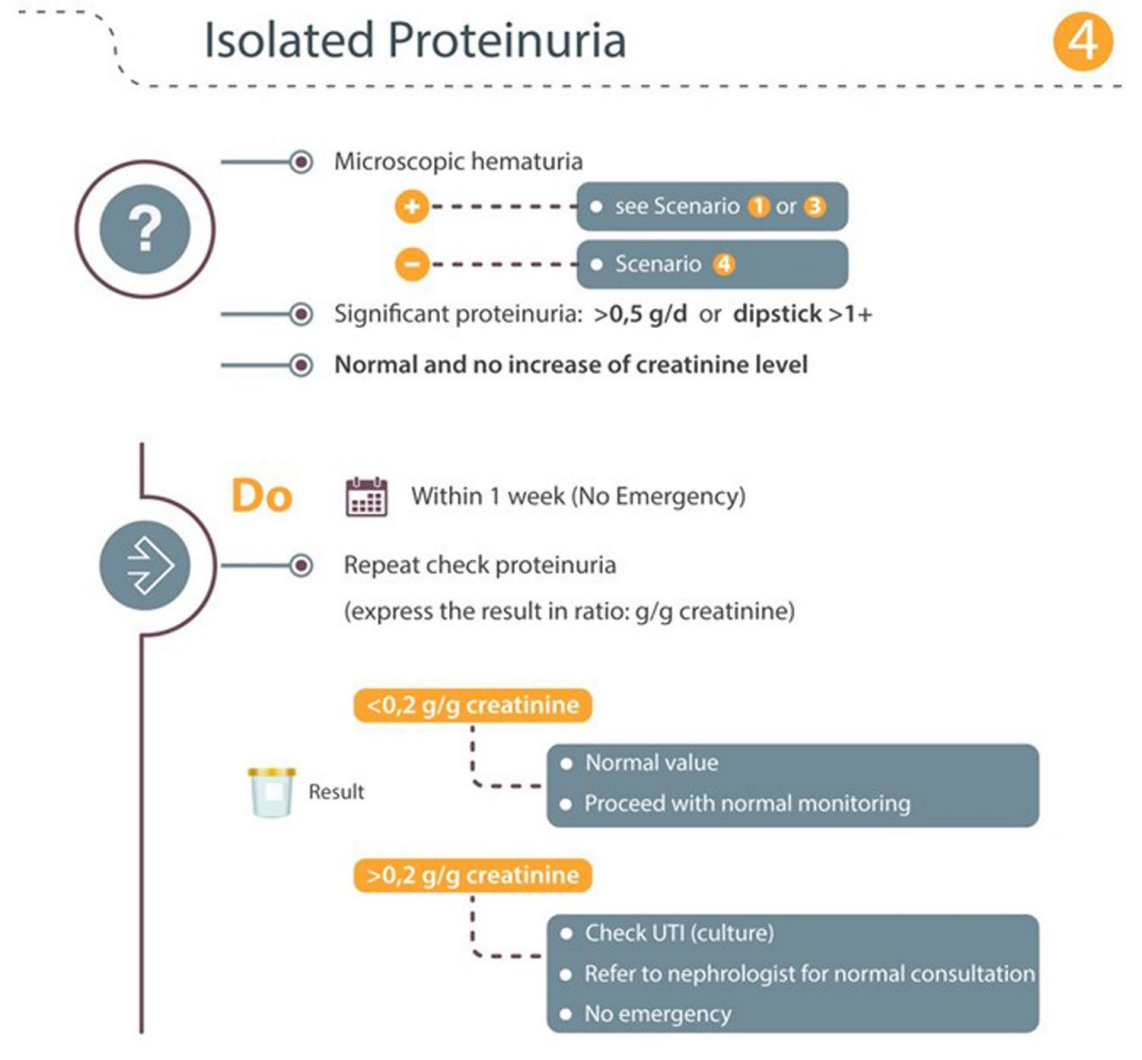
Isolated proteinuria

When significant proteinuria is detected (> 0.5 g/day or on dipstick > 1 +), we recommend determining proteinuria on a random urine sample and have proteinuria expressed in g/g creatinine (Fig. 4). A result for proteinuria below 0.2 g/g creatinine is normal and does not need further investigations. In this circumstance, normal monthly monitoring should be resumed. A result for proteinuria above 0.2 g/g creatinine needs further investigation. When a UTI is excluded, patients with pathologic proteinuria should be referred to a nephrologist for a non-urgent consultation.

### Scenario 5: Subjects with renal symptoms

**Fig. 5:**
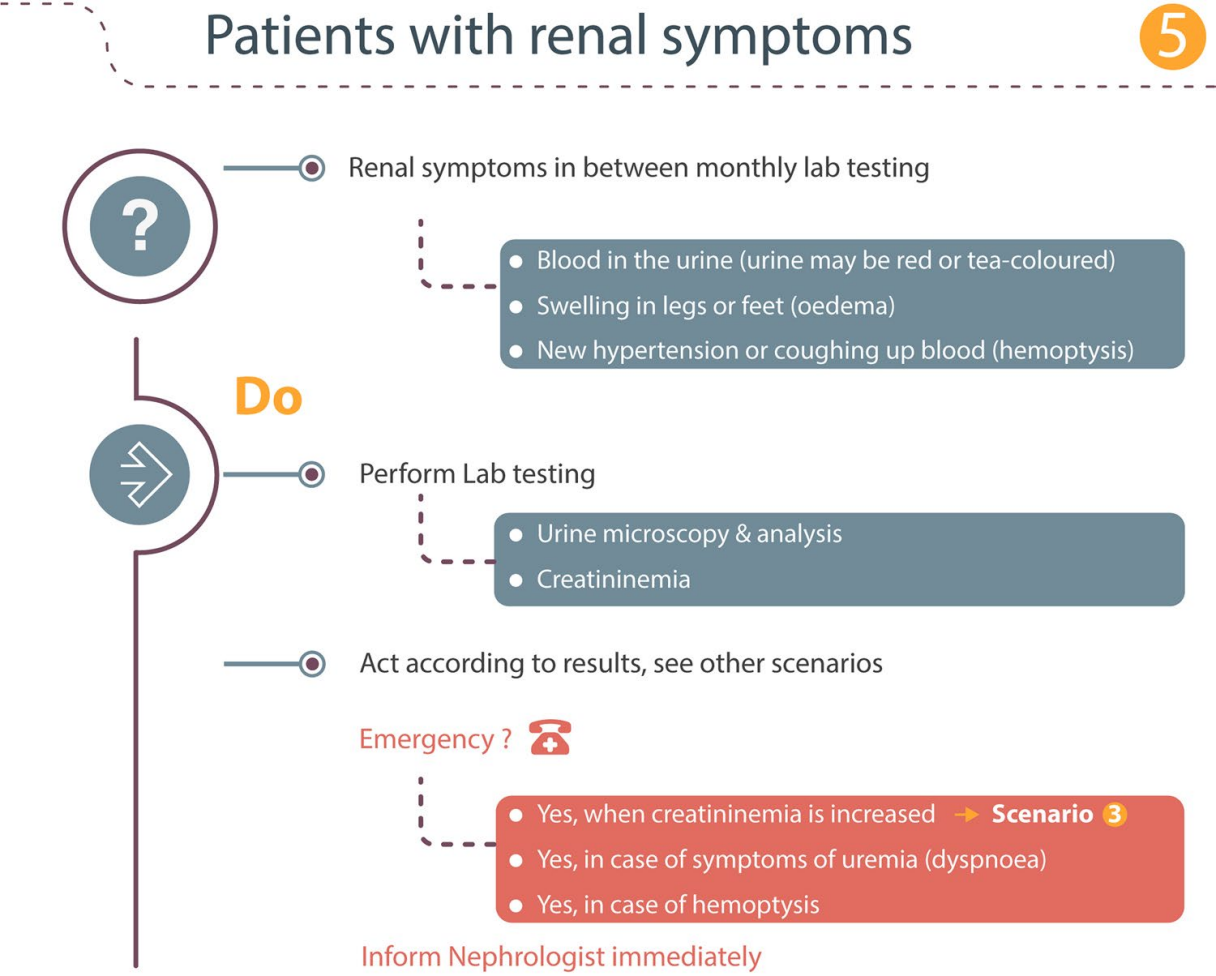
Renal symptoms

Subjects treated with alemtuzumab are educated to be vigilant for symptoms of possible kidney disorders (Fig. 5). These include red-discoloration of the urine and swelling of the legs, feet, or eyelids. In addition, coughing up blood is an alarm symptom for which patients should immediately contact their neurologist or seek immediate medical attention as it may signal underlying pulmonary damage/bleeding [6]. When a patient observes such symptoms in between the monthly lab testing, the following approach is proposed:

1. Perform lab testing on urine and blood.
2. Act according to the results as described in the other scenarios.

### Scenario 6: Isolated elevated serum creatinine

**Fig. 6:**
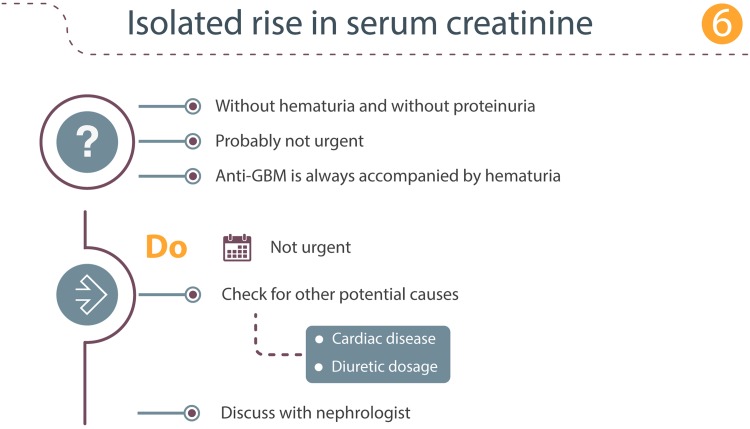
Isolated rise in serum creatinine

The finding of an elevated serum creatinine without hematuria or proteinuria is unlikely to require immediate action (Fig. 6). Anti-GBM disease is always accompanied with hematuria, and the predominant presentation of MN is proteinuria. Of course, it is important to evaluate the patient for other causes of increased serum creatinine such as reduced fluid intake, (undetected) cardiac disease, or unadjusted diuretics dosage. A non-urgent consultation with a nephrologist is advised.

## Discussion

The use of alemtuzumab has been associated, albeit rarely, with the development of immune-mediated renal adverse events. Membranous nephropathy is not a nephrologic emergency, but requires intensive follow-up and treatment. Anti-GBM disease is potential cause of life-threatening acute renal failure and requires immediate treatment. Anti-GBM disease following alemtuzumab therapy in multiple sclerosis (MS) patients was first reported in 2006 [40]. In response, Sanofi Genzyme implemented mitigation strategies in the clinical development program. In the post marketing setting, according to the label, a monthly blood and urine analysis until 48 months after the last alemtuzumab infusion is mandatory to capture potential autoimmune AEs in their preclinical stage.

Initial treatment of membranous nephropathy is symptomatic and directed at the reduction of edema and proteinuria. In patients with renal abnormalities and positive anti-GBM antibodies, this treatment should be initiated without delay. The standard treatment of anti-GBM disease consists of plasmapheresis, cyclophosphamide, and steroids.

In this consensus article, we have presented six different scenarios of renal abnormalities that may be encountered and the appropriate actions to take for each scenario.

## Conclusion

The use of alemtuzumab has been associated with the development of renal immune-mediated adverse events in 0.3% of patients in clinical trials in MS, which generally occurred within 39 months of the last administration. Through the implementation of the risk minimisation measures, patients can be diagnosed, and treated if needed, early allowing for generally favorable outcomes. This important goal can be reached through HCP and patient education, careful analysis of the monthly lab tests, and close collaboration between the patient, neurologist, and the nephrologist. Moreover, collaboration between the neurologist and nephrologist could be facilitated through the pre-establishment of a physician reference network. In this report, we have described different scenarios of renal abnormalities that may be encountered following the administration of alemtuzumab to RRMS patients, and the appropriate actions to take for each.
